# Fructose-Based Acrylic Copolymers by Emulsion Polymerization

**DOI:** 10.3390/polym10050488

**Published:** 2018-05-02

**Authors:** Jessica S. Desport, Mónica Moreno, María J. Barandiaran

**Affiliations:** POLYMAT, University of the Basque Country UPV/EHU, 20018 Donostia-San Sebastián, Spain; jessica.desport@ehu.eus (J.S.D.); monica.moreno@polymat.eu (M.M.)

**Keywords:** fructose, monosaccharide, carbohydrates, renewable resources, emulsion polymerization

## Abstract

The exploration of a renewable resource for the preparation of waterborne copolymers was conducted. Low molar mass sugar resources were selected for their wide availability. A fructose-based monomer (MF) bearing a methacrylate radically polymerizable group was successfully synthesized. The latter was shown to be able to homopolymerize in emulsion. The high *T*_g_ of the resulting polymer (about 115 °C) makes it of particular interest for adhesive and coating applications where hard materials are necessary to ensure valuable properties. As a result, its incorporation in waterborne acrylic containing formulations as an equivalent to petrochemical-based methyl methacrylate was investigated. It was found that the bio-based monomer exhibited similar behavior to that of common methacrylates, as shown by polymerization kinetics and particle size evolution. Furthermore, the homogeneous incorporation of the sugar units into the acrylate chains was confirmed by a unique glass transition temperature in differential scanning calorimeter (DSC). The potential of MF for the production of waterborne copolymers was greatly valued by the successful increase of formulation solids content up to 45 wt %. Interestingly, polymer insolubility in tetrahydrofurane increased with time due to further reactions occurring in storage. Most likely, the partial deprotection of sugar units was the reason for the creation of hydrogen bonding and, thus, physically insoluble entangled chains. This behavior highlights opportunities to make use of hydroxyl groups either for further functionalization or, eventually, for achieving enhanced adhesion on casted substrates.

## 1. Introduction

The increasing environmental regulations and strong public concern have motivated the polymer industry to move towards more environmentally friendly processes, and emulsion polymerization is, nowadays, one of the most widespread green strategies. However, these waterborne processes relied mostly on petroleum-based chemistry. This approach is non-sustainable because the feedstocks need millions of years to be formed. Therefore, the search for alternatives based on renewable resource monomers for the production of novel polymers able to substitute their petroleum-based counterparts is eagerly sought. These polymer classes may contribute to reducing the environmental impact of polymer production by reducing the carbon footprint [1].

The potential to use either natural polymers, such as casein [2], or monomers from biomass feedstocks, such as fatty acid derivatives [3,4], as raw materials to produce waterborne latexes for industrial applications has already been demonstrated. On the other hand, carbohydrates represent a rich class of natural products in terms of availability, functionality, and molecular weights. Indeed, carbohydrates represent about 75% of the annual renewable biomass (200 billon ton approximately), with only around 4% used by humans [5]. Moreover, a substantial part of the carbohydrates commercially available in the food industry are regrettably wasted as a surplus of their agricultural production or through domestic wastage. The valorization of those food wastes appears more relevant than ever. In this context, the use of low molecular weight carbohydrates to produce bio-based monomers able to polymerize through free radical polymerization in aqueous dispersed media emerges as a promising approach towards sustainable development. However, their raw structure, which mostly consists of alcoholic functions, does not allow their direct use as monomers in conventional radical polymerization. The presence of a reactive vinyl or (meth)acrylic group in the monomer is required. Moreover, the molecule has to be hydrophobic enough to be polymerized in aqueous dispersed media. This requires the synthesis of well-defined monofunctionalized (meth)acrylate hydrophobic saccharides.

Indeed, the use of hydrophobic sugar monomers in emulsion polymerization has not received much attention. Only a few examples can be cited, and all of them are based on the protected methacryloyl glucose (3-*O*-methacryloyl-1,1,5,6-diisopropylydene-d-glucose or 3-MDG). In the 80s, Klein et al. [6] investigated the emulsion polymerization of 3-MDG at solids content of up to 15 wt %. Using non-ionic emulsifiers, they stated conversions of up to 92%. Forcada et al. [7,8] studied the emulsion copolymerization of 3-MDG for the production of microgels for biomedical applications. Takasu et al. [9] investigated the seeded emulsion copolymerization of VAc with vinyl sugars and focused their investigations on the resulting accelerated rate of biodegradation of the material. On the other hand, Yaacoub and co-workers widely investigated the 3-MDG homopolymerization in emulsion at low solids content from 5 to 20 wt % [10]. The same group studied the batch [11] emulsion copolymerization of 3-MDG with butyl acrylate at 10 wt % solids content and subsequently succeeded in increasing the solids content up to 50 wt % with reasonable coagulum amounts by using semi-continuous processes [12,13,14]. They also extended their investigations to free radical [15] and RAFT mini-emulsion copolymerization [16].

In this work, and to further expand the range of application of those carbohydrates, the use of fructose as the renewable monosaccharide source for the production of bio-sourced monomers and waterborne polymers has been explored. Indeed, fructose can be obtained at an industrial scale from fruit waste. Nevertheless, very rare examples of the use of fructose as building blocks for bio-based polymers can be found in literature. Methacrylate fructose was cited as a potentially interesting monomer for the production of waterborne coatings [17]. However, to the best of our knowledge, actual polymerization results have not been reported yet. More recently, methacrylate fructose was used to synthesize nanocarriers via RAFT polymerization for biomedical applications [18].

For the purpose of this research, fructose-based monosaccharide bearing acetonide protecting groups and being commercially available was envisaged as valuable precursor. This structure was selected because it presents a primary alcohol situated in the anomeric position, which allows high selectivity in terms of functionalization, as well as high reactivity towards a common nucleophile. The incorporation of a methacrylate polymerizable group in the above-mentioned precursor was investigated first. Next, the potential use of the resulting product as a substitute of petrochemical raw materials commonly used in waterborne acrylic containing formulations—typically used as adhesives or coatings—was explored. As a first approach, low solids content emulsion polymerization with different sugar-based monomer/butyl acrylate (BA) compositions was studied. Taking advantage of the understanding gained in this first part, the study was extended to high solids content systems, which are very important for effective industrial applications. In all the cases, polymerization kinetics, polymer characteristics, thermal properties, and the quality of the resulting films are reported and discussed.

## 2. Materials and Methods

### 2.1. Materials

All reactants were used without prior purification. 2,3:4,5-Di-*O*-isopropylidene-b-d-fructopyranose, also called diacetone-d-fructose (DAF, >98%), was obtained from Carbosynth (Compton, UK). Butyl acrylate (BA, 99.5%) was purchased from Quimidroga (Barcelona, Spain). Methacrylic anhydride (94%), triethylamine (NEt3, >99.5%), 4-dimethylaminopyridine (DMAP, >99%), potassium persulfate (KPS, >99%), sodium dodecyl sulphate (SDS, >98.5%), and NaHCO_3_ (99.7%) were from Sigma-Aldrich (Steinheim, Germany). Solvents were purchased from Acros-Organics (Geel, Belgium) and Sigma-Aldrich.

### 2.2. Synthesis of the Diacetone-d-Fructose Monomer

**Scheme 1:**
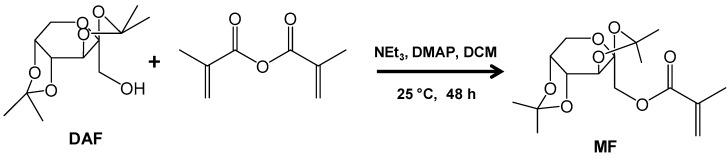
Functionalization of protected fructose in solvent at ambient temperature.

The transesterification reaction was done in dichloromethane (DCM) at an ambient temperature by mixing DAF (1 eq) and methacrylic anhydride (1 eq) in the presence of NEt_3_ and DMAP (0.2 eq), as catalysts, under a nitrogen atmosphere (Scheme 1).

Product isolation was performed through extraction in DCM, drying over Na_2_SO_4_, and solvent evaporation. This was followed by column chromatography, obtaining a 85 wt % yield. The resulting methacrylate protected fructose monomer (named MF hereafter) has a melting point in the range of 30–35 °C.

### 2.3. Emulsion Polymerization

Batch emulsion polymerization of MF was performed in a 250 mL double wall glass reactor, equipped with a turbine stirrer, a nitrogen inlet, a reflux condenser, a temperature probe, and a sample device. The reaction temperature was controlled by a Lauda water bath. The sugar-based monomer was heated up and introduced as a melted substance in the reactor containing an aqueous phase made of deionized water, emulsifier (SDS, 4 %wbm), and buffer (NaHCO_3_, 4 %wbm) pre-heated at 60 °C. The pre-emulsion was prepared under nitrogen purging and strong agitation stirring (800 rpm). Then, the temperature was increased to 70 °C and the initiator (KPS, 2 %wbm), previously dissolved in water, was introduced in the media. A low solids content of 10 wt % was targeted.

Emulsion copolymerization reactions were carried out in batches by varying the bio-based monomer content and the initiator amount in some cases. A 30 wt % solids content, as well as 45 wt % solids latexes, were synthesized. For the preparation of the emulsion, the organic phase composed by the monomer mixture (MF and BA) and the aqueous phase containing the emulsifier and the buffer, used to avoid drops in pH during the reaction, were mixed under magnetic stirring (15 min at 900 rpm). The resultant mixture was poured in a double wall glass reactor equipped with a condenser, a nitrogen inlet, a sample device and a stainless steel turbine stirrer. Reactions were carried out at 70 °C under agitation (250 rpm) at a pH of approximately 9. The initiator KPS was injected as a shot when the pre-emulsion temperature reached 70 °C, defining the beginning of the reaction. The system was then allowed to react in batches. Samples were withdrawn at regular intervals and the inhibitor (hydroquinone) was used to stop reaction and allow monomer conversion follow-up time, which was performed by ^1^H-NMR. A standard formulation for the synthesis of 30 wt % solids content copolymers included 2 %wbm SDS, 1 %wbm KPS, and 1.5 %wbm NaHCO_3._

The copolymer composition in terms of both the weight and the molar fraction of the investigated latexes, together with the nomenclature to be used throughout this manuscript, is reported in Table 1. The first number of the latex name refers to the weight percentage of the sugar-based monomer while the second number corresponds to the solids content.

### 2.4. Characterization

Samples were withdrawn during a polymerization reaction and ^1^H-NMR spectroscopy was used to monitor monomer conversion. NMR sample preparation was carried out with 50 µL of latex and 450 µL of a solution containing benzene-1,3,5-tricarboxylic acid (BTC) as a reference at a concentration of 1 g/L. ^1^H-NMR spectra were recorded on Bruker 400 MHz equipment, using the WATERGATE method to suppress the signal of water, and theintegration of the vinyl proton signals and reference signal was performed.

The Z-average particle diameter was determined using dynamic light scattering (DLS, Zetasizer Nano Z, Malvern Instruments, Malvern, UK). Latexes were diluted with deionized water before measurements.

The gel content was determined by Soxhlet extraction of the dried polymer with tetrahydrofuran (THF) under reflux for 24 h, and it is reported as the ratio between the weight of the insoluble polymer fraction and that of the initial sample.

The glass transition temperature (*T*_g_) of the polymers was measured through means of a differential scanning calorimeter, series DSC Q1000 (TA Instruments, New Castle, DE, USA). The measurements were carried out in a range from −70 to 200 °C at a heating rate of 10 °C/min. For thermal resistance measurements, a thermogravimetric analyzer TGA 2950/Q500 TA instrument was used, and the experiments were carried out with a heating rate of 10 °C/min from room temperature to 800 °C.

## 3. Results

### 3.1. Fructose-Based Monomers

**Figure 1:**
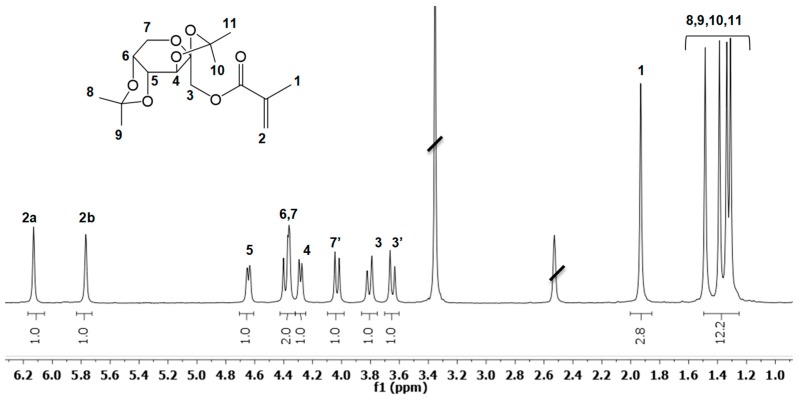
^1^H-NMR spectrum of the methacrylate protected fructose (MF) monomer in DMSO-*d*_6_.

Figure 1 reports the proton ^1^H-NMR spectrum of the pure resulting monomer. The methacrylate functional group is recognizable: vinyl protons are giving signals at 5.8 and 6.2 ppm and methyl protons appear as one singlet at 1.9 ppm. The presence of the sugar ring protons is confirmed by the signals between 3.5 and 4.8 ppm.

**Figure 2:**
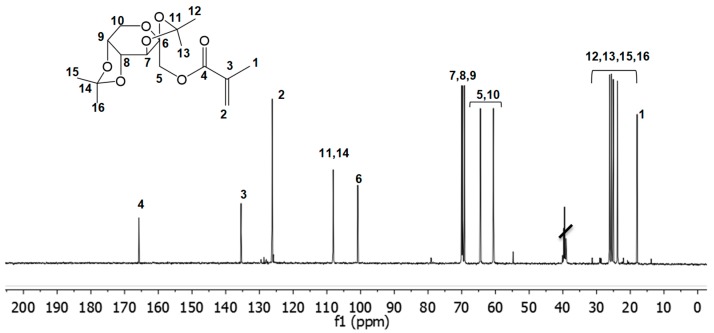
^13^C-NMR spectrum of the methacrylate protected fructose (MF) monomer in DMSO-*d*_6_.

The ^13^C-NMR spectrum given in Figure 2 corroborates these results by highlighting a structure bearing 16 well identified carbons.

### 3.2. Homopolymer and Its Thermal Characterization

Full conversion was achieved in the emulsion homopolymerization of MF, as the complete disappearance of the vinyl proton signals was observed after 1 h of the reaction with ^1^H-NMR. Moreover, small particle sizes (67 nm) were obtained, and the molecular weight (1 × 10^6^ Da) was in the range of emulsion polymers. The homopolymer exhibited a high glass transition temperature of about 115 °C, which can be explained by the cyclic nature of the sugar combined with the rigidity of the diacetonide protecting groups, which limits the mobility amplitude of the chains. Because of the high *T*_g_, this monomer appears as a potential equivalent to the petrochemical methyl methacrylate raw material. For detailed ^1^H-NMR, GPC chart, and DSC see the Supporting Information section.

### 3.3. Fructose-Based Emulsion Copolymerization

**Figure 3:**
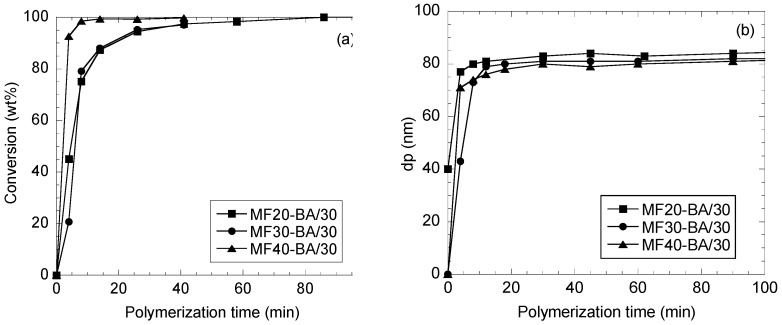
Methacrylate fructose-based copolymers. (**a**) Kinetics and (**b**) particle size evolution for copolymerizations carried out with 1.0 wbm% of KPS.

Figure 3 presents the kinetics and the particle size evolution of the different copolymerizations carried out with the methacrylate fructose and BA at 30% solids content and 1 wbm% of KPS. All reactions occurred very fast and no differences among the different compositions were observed, except for the faster kinetics of the MF40-BA/30.

**Figure 4:**
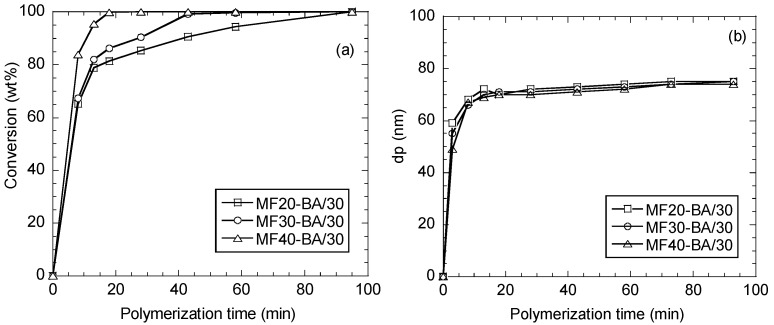
Methacrylate fructose-based copolymers. (**a**) Kinetics and (**b**) particle size evolution for copolymerizations carried out with 0.5 wbm% of KPS.

In order to clarify the behavior of MF40-BA/30, another set of reactions using a lower amount of initiator (0.5 wbm%) were performed (Figure 4). As expected, slower kinetics were observed but, again, the reaction containing 40 wt % of MF was the fastest. It can be speculated that due to the much higher reactivity of the methacrylates with respect to BA, the effect of the composition on the overall conversion would be more pronounced when dealing with a larger methacrylate composition [19].

#### 3.3.1. Polymer Characterization

**Figure 5:**
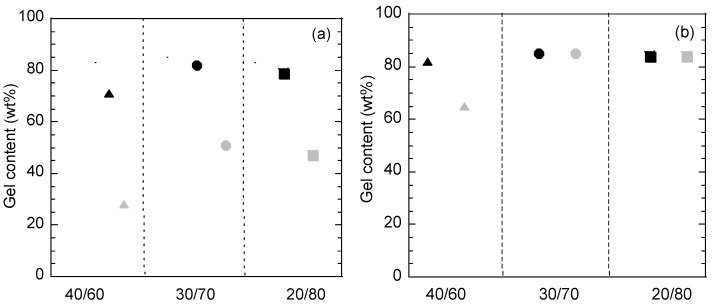
The gel content of sugar-based latexes prepared with different MF/BA ratios at 1 wbm% KPS (▲●■) and 0.5 wbm% KPS (▲●■). (**a**) Initial measurements; (**b**) Measurements repeated after 15 days.

The gel content (an insoluble polymer in THF) was measured using Soxhlet extraction. Large amounts of gel were obtained in all cases, as shown in Figure 5. On one hand, a large amount of gel is expected when dealing with large BA content latexes. This is because of intermolecular chain transfer to polymer followed by termination through combination, as reported in literature [20]. However, it is also mentioned that the incorporation of methyl methacrylate (MMA) units in butyl acrylate chains promotes the decrease of gel content [21]. Indeed, MMA terminated chains are less reactive towards hydrogen abstraction and the absence of abstractable hydrogens in the MMA units, together with the fact that MMA radicals terminate predominantly by disproportionation, explain the significant reduction of gel content. Thus, since the bio-based monomer is carrying a methacrylate function, a decrease in gel could be expected as more methacrylate sugar is incorporated. This effect was observed for the two sets of reactions involving MF/BA copolymerization, i.e., using both 0.5 wbm% KPS and 1% KPS. Additionally, less gel content was found when a smaller amount of KPS was employed. Latexes containing 40% of methacrylate fructose exhibited a gel content of 28% when prepared with 0.5 wbm% KPS and a gel content of 72% when prepared with 1 wbm% KPS (Figure 5a). This unexpected result goes against theory that a larger initiator concentration induces a superior number of radicals per particle, promoting termination reactions and, hence, lower gel contents.

However, it is important to note that the measurement of the gel was not performed systematically but after few days the latex was synthesized. Indeed, the gel presented in Figure 5a corresponding to 1 wbm% KPS was measured after 26 days of its synthesis. Meanwhile, the gel with 0.5 wbm% KPS was measured just after 3 days of storage. Thus, the unexpected lower gel amount for 0.5 wbm% KPS latexes could be attributed to some evolution of the latex during storage. In fact, recent studies in our lab have shown that the gel content of MMA/BA (50/50) latex increases during storage when the latex does not contain hydroquinone (HQ), likely because of the presence of tertiary radicals which are very stable and can survive long time. In our case, the latexes were stored without HQ. Therefore, this hypothesis could help explain these results.

Another aspect to take into consideration is the probability of sugar side chains to suffer partial deprotection, yielding –OH groups that could aggregate due to hydrogen bonding. It is well known that the isopropylidene protecting groups can be cleaved under acidic conditions [22]. However, it was reported that protecting groups within non-water soluble polymer chains are rather hard to cleave. Bird et al. [23] detailed the particular resistance to deprotection of methacrylate galactose homopolymers, showing that heating at 100 °C in HCl for several hours failed in deprotecting the carbohydrate units. In our work, reactions were performed at a basic pH by using NaHCO_3_ as buffer, so that hydrolysis of the protecting groups was not favored. In any case, it is worth noting that if the polymer chains have a significant molecular weight, just a few –OH in the chain would be enough to lead to association by hydrogen bonding and to form polymer chains that are not soluble in THF.

To verify if the latexes evolved during storage, gel measurement of the latexes were repeated 15 days later. The copolymer were 18 days and 41 days old at this point (latexes synthesized with 0.5 wbm% KPS and 1 wbm% KPS respectively). Figure 5b summarizes the results. A clear increase of gel was observed after longer storage for all the latexes of the 0.5 wbm% KPS series. On the other hand, the comparison of the 1 wbm% KPS series also shows that the latex is evolving during storage. From the observed evolution, it can be concluded that the gel fraction of the three series are comparable and that the insoluble fraction in THF most likely increases with time because of a little deprotection of the sugar chains and hydrogen bond formation.

#### 3.3.2. Thermal Characterization and Film Properties

The glass transition temperatures of the resulting MF/BA copolymers were measured using DSC. It was observed that all copolymers exhibited one single T_g_ (curves are given in Supporting Information) and, as revealed in Table 2, the values were in good agreement with those predicted by the Fox equation (Equation (1)),

(1) $$\frac{1}{T_{g}}=\frac{w_{1}}{T_{g1}}+\frac{w_{2}}{T_{g2}}$$

where w_1_ and w_2_ are the weight fractions of MF and BA, considering the T_g_ of the sugar-based homopolymer at 115 °C and that of the BA at −50 °C.

**Figure 6:**
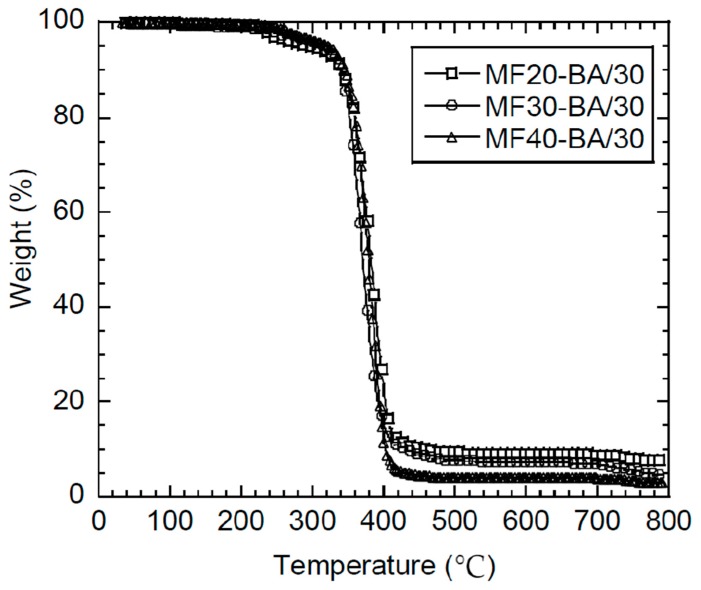
Thermogravimetric analysis of copolymers MF/BA.

The thermal stability of the different sugar-based copolymers was evaluated by thermogravimetric analysis. It can be seen in Figure 6 that thermal stability was independent from the carbohydrate-monomer content. The copolymers exhibited very good thermal stability, since no significant degradation was observed below 350 °C. Moreover, copolymer degradation was achieved in a single step, suggesting homogeneity in chain composition.

**Figure 7:**
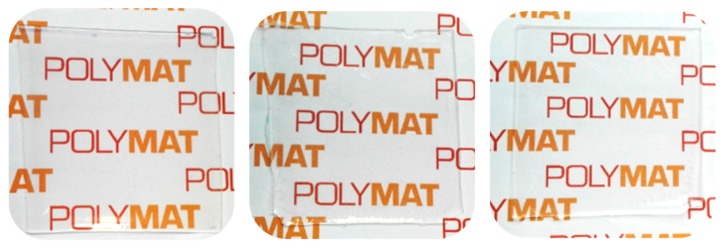
Pictures of polymer films containing (from left to right) 20, 30, or 40 wbm% methacrylate fructose.

The visual appearance of films casted on silicon moulds at ambient temperature and 55% humidity is given in Figure 7. In all cases, continuous transparent films were obtained. This result was in accordance with the thermal properties of the copolymer shown above, indicating a good incorporation of the sugar units in butyl acrylate chains.

### 3.4. 45 wt % Solids Content Sugar-Based Copolymer Latexes

**Figure 8:**
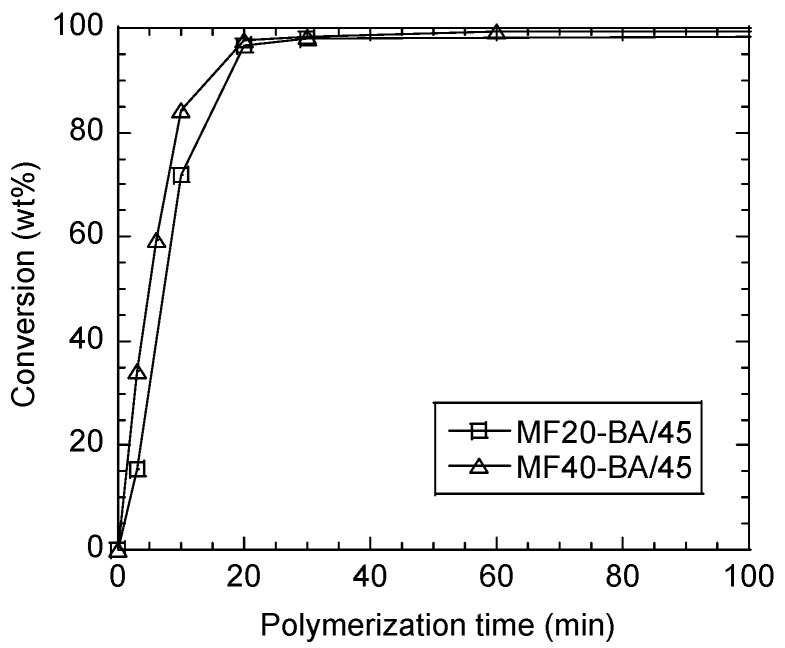
The evolution of overall conversion for the copolymerization of MF with BA at different ratios and 45% solids content.

30 wt % solids content latexes prepared from sugar-based methacrylate monomers presented excellent colloidal and thermal stability, as well as a homogeneous incorporation of the sugar units in butyl acrylate chains. However, it was not possible to properly cast thin films because of wettability issues. Latexes tend to shrink when applied on various substrates such as glass, metal, or plastic. Moreover, since high solids content latexes are sought for industrial applications, the formulation was modified to produce 45 wt % solids content latexes. To enhance electrostatic stabilization of the particles, a disulfonate carrying emulsifier (Dowfax 2A1, 2 wbm%) was used. Acrylamide is a water-soluble monomer which can generate oligomers or polymers in the aqueous phase, causing more interactions and resulting in an increase in viscosity. Raising viscosity helps have better wetting when casting films; also, amide functions are well-known to promote adhesion [24]. Thus, 2 wbm% of acrylamide was added to the formulation, and 0.75 wbm% of KPS plus 1 wbm% NaHCO_3_ was used. The latexes made were very stable, with final average particle sizes of approximately 110 nm and having a narrow distribution (Ð < 0.04; Ð = (width/mean)² in DLS Malvern equipment). Particle size distributions can be found in Supporting Information. The evolution of the reaction was also very fast, as exemplified in Figure 8.

Excellent wettability features were observed on different substrates, such as plastic, glass, and metal, and the resulting films were continuous and transparent. A summary of the final copolymer characteristics is given in Table 3. In this case, rather large amounts of gel fraction were also obtained. Values around 80% were measured independent of monomer type and composition. It is important to note that Soxhlet extractions were carried out after several days of storage.

## 4. Conclusions

A methacrylate fructose monomer bearing acetonide protecting groups was synthesized from a commercially available protected fructose-based monosaccharide. The resulting homopolymer presented a *T*_g_ of approximately 115 °C, appearing as an interesting alternative to petrochemical-based methyl methacrylate, which is commonly used in waterborne acrylic formulations for adhesives and coatings.

The copolymerization of fructose-based monomers with butyl acrylate in batch emulsion polymerization was investigated. High conversions were achieved in all cases, and the polymer particle size was independent from the bio-based monomer content. It was observed that gel was evolving during storage, likely because of some deprotection of the sugar chains and hydrogen bond formation. Furthermore, latexes led to continuous transparent films with a unique glass transition temperature, which was in the range of the theoretical value calculated from the Fox equation. The films also had excellent thermal stability. The potential of the sugar-based monomer for the production of waterborne copolymers was greatly evident through the successful increase of solids content in the formulation to up to 45%, which is important for effective industrial applications.

## Figures and Tables

**Table 1:** Studied methacrylate sugar-based copolymer compositions (in terms of both weight and molar fractions) and their nomenclature.

Latex Name		MF/BA (wt %)	MF/BA (mol %)
Low Solids	High Solids		
**MF20-BA/30**	**MF20-BA/45**	20/80	9/91
**MF30-BA/30**	**MF30-BA/45**	30/70	14/86
**MF40-BA/30**	**MF40-BA/45**	40/60	21/79

**Table 2:** Measured and predicted *T*_g_ of the different methacrylate sugar-based copolymers.

Monomer Composition MF/BA	20/80	30/70	40/60
**T_g_ (°C) measured**	−30	−25	−2
**T_g_ (°C) Fox-equation prediction**	−29	−17	−4

**Table 3:** Final copolymer characteristics.

Run	dp (nm)	T_g_ (°C)	Gel (wt %)
**MF20-BA/45**	107	−24	76
**MF40-BA/45**	106	3	75

## Supplementary Materials

The following are available online at http://www.mdpi.com/2073-4360/10/5/488/s1. Figure S1: ^1^H-NMR spectrum of the MF homopolymer in D_2_O. WATERGATE method. Figure S2: Molecular weight distribution of the MF homopolymer. Figure S3: DSC of the MF homopolymer., Figure S4: Particle size distribution of different copolymers: (**a**) MF40-BA/30, with 1% KPS; (**b**) MF40-BA/30, with 0.5% KPS; and (**c**) MF40-BA/45. Figure S5: DSCs of MF/BA copolymers of different compositions: (**a**) MF20-BA; (**b**) MF30-BA; and (**c**) MF40-BA.
