# Supplementary material for: Fructose-Based Acrylic Copolymers by Emulsion Polymerization
Source: Polymers (Basel). 2018 May 2;10(5):488. doi: 10.3390/polym10050488 (PMC6415512; doi:10.3390/polym10050488)
Supplement: Supplementary file 1 [file polymers-10-00488-s001.pdf]

polymers

MDPI

Supporting Information

Fructose-based acrylic copolymers by emulsion polymerization

Jessica S. Desport[1], Mónica Moreno[1], María J. Barandiaran[1,*]

[1] POLYMAT, University of the Basque Country UPV/EHU, 20018 Donostia-San Sebastián, Spain jessica.desport@ehu.eus (J.D.); monica.moreno@polymat.eu (M.M.); mariaje.barandiaran@ehu.eus (M.J.B)

* Correspondence: mariaje.barandiaran@ehu.eus; Tel.: +34-943-015330

Received: date; Accepted: date; Published: date

Supplementary Materials: The following are available online at www.mdpi.com/link, Figure S1: ^{1}H-NMR spectrum of the MF homopolymer in D_2O. WATERGATE method, Figure S2: Molecular weight distribution of the MF homopolymer, Figure S3: DSC of the MF homopolymer, Figure S4: Particle size distribution of different copolymers: (a) MF40-BA/30, with 1% KPS; (b) MF40-BA/30, with 0.5% KPS; (c) MF40-BA/45, Figure S5: DSCs of MF/BA copolymers of different composition: (a) MF20-BA; (b) MF30-BA; (c) MF40-BA

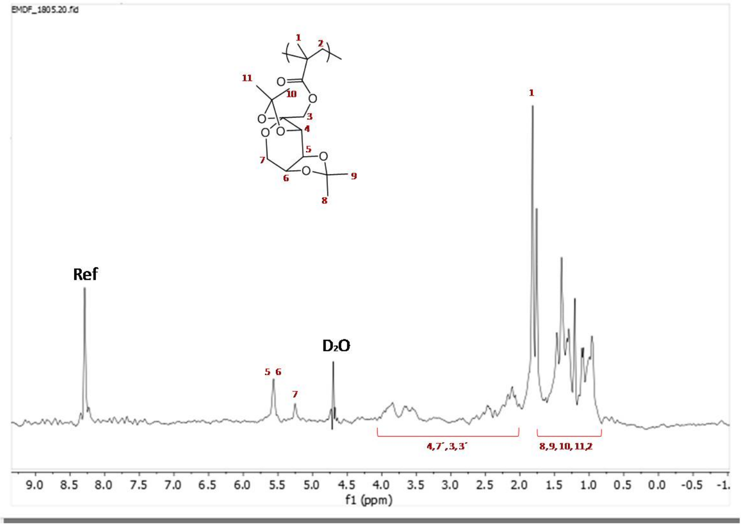

Figure S1. ^{1}H-NMR spectrum of the MF homopolymer in D_2O. WATERGATE method.

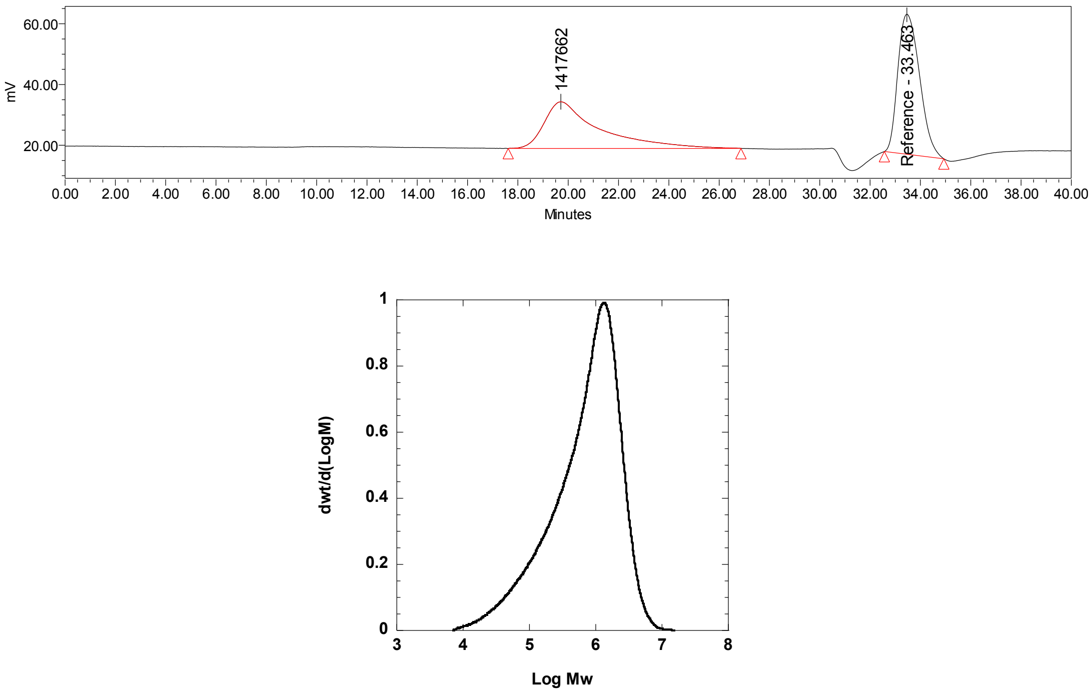

Figure S2: Molecular weight distribution of the MF homopolymer. Agilent technologies GPC system, equipped with 3 Shodex columns, refractive index and UV detectors. THF was used as solvent. The analysis was carried out at 1.0 mL/min flow rate. Relative molecular weight was determined by means of a conventional calibration obtained with polystyrene narrow standards.

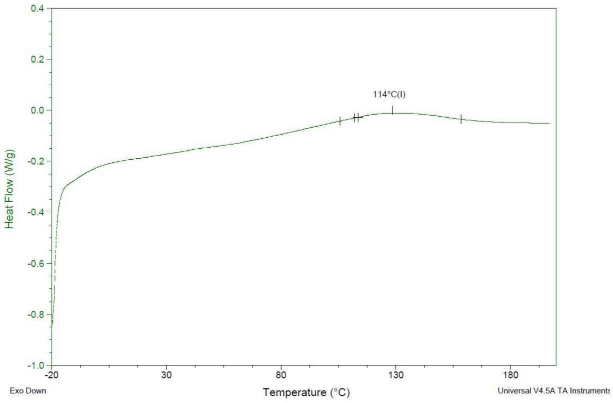

Figure S3: DSC of the MF homopolymer

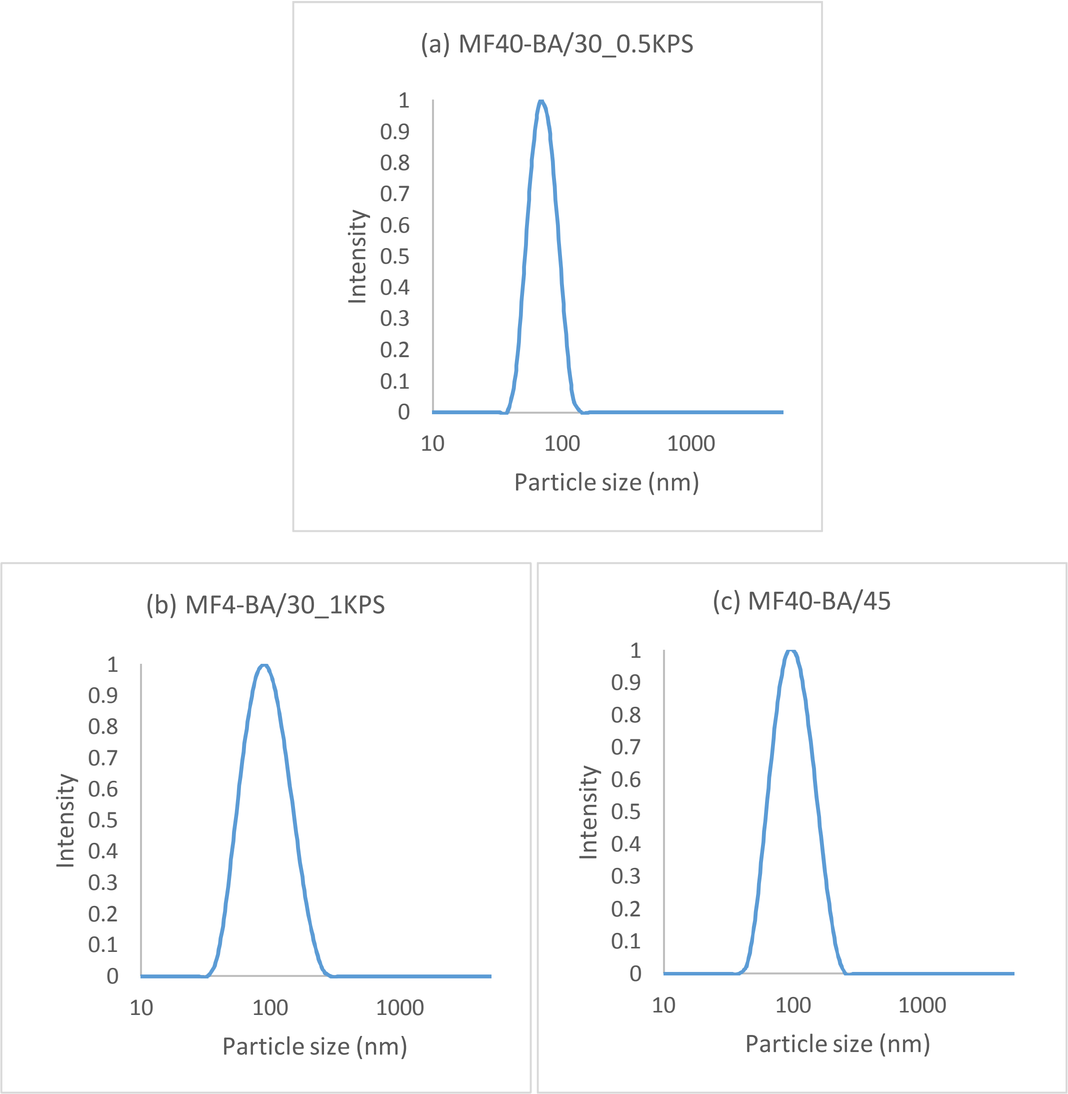

Figure S4: Particle size distribution of different copolymers: (a) MF40-BA/30, with 1% KPS; (b) MF40-BA/30, with 0.5% KPS; (c) MF40-BA/45

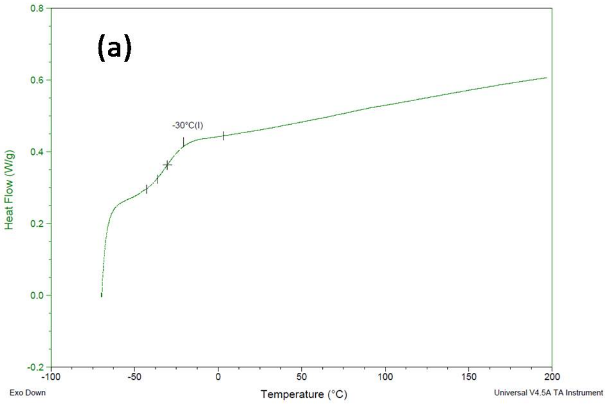

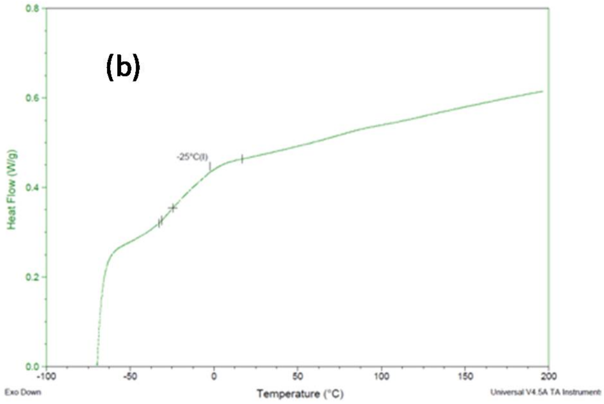

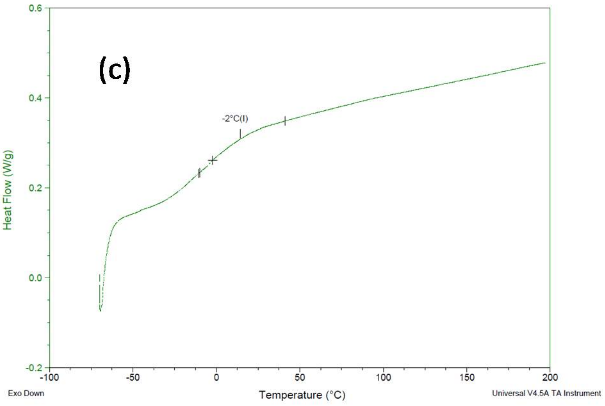

Figure S5: DSCs of the different copolymers: (a) MF20-BA; (b) MF30-BA; (c) MF40-BA

(http://creativecommons.org/licenses/by/4.0/).
